# Establishment of a novel culture method for maintaining intestinal stem cells derived from human induced pluripotent stem cells

**DOI:** 10.1242/bio.049064

**Published:** 2020-01-09

**Authors:** Satoshi Kondo, Shota Mizuno, Tadahiro Hashita, Takahiro Iwao, Tamihide Matsunaga

**Affiliations:** 1 Department of Drug Safety Research, Nonclinical Research Center, Tokushima Research Institute, Otsuka Pharmaceutical Co., Ltd., Tokushima, Japan; 2 Department of Clinical Pharmacy, Graduate School of Pharmaceutical Sciences, Nagoya City University, Nagoya, Japan

**Keywords:** Human iPS cells, Intestinal stem cells, Pharmacokinetics

## Abstract

The small intestine plays an important role in the pharmacokinetics of orally administered drugs due to the presence of drug transporters and drug-metabolizing enzymes. However, few appropriate methods exist to investigate intestinal pharmacokinetics. Induced pluripotent stem (iPS) cells can form various types of cells and represent a potentially useful tool for drug discovery. We previously reported that differentiated enterocytes from human iPS cells are useful for pharmacokinetic studies; however, the process is time and resource intensive. Here, we established a new two-dimensional culture method for maintaining human iPS-cell-derived intestinal stem cells (ISCs) with differentiation potency and evaluated their ability to differentiate into enterocytes exhibiting appropriate pharmacokinetic function. The culture method used several factors to activate signalling pathways required for maintaining stemness, followed by differentiation into enterocytes. Functional evaluation was carried out to verify epithelial-marker expression and inducibility and activity of metabolic enzymes and transporters. Our results confirmed the establishment of an ISC culture method for maintaining stemness and verified that the differentiated enterocytes from the maintained ISCs demonstrated proper pharmacokinetic function. Thus, our findings describe a time- and cost-effective approach that can be used as a general evaluation tool for evaluating intestinal pharmacokinetics.

## Abstract (precis)

**Summary:** This study describes a novel two-dimensional culture method for maintaining intestinal stem cells (ISCs) derived from human iPS cells. The enterocytes differentiated from maintained ISCs showed pharmacokinetics functions.

## INTRODUCTION

Intestinal enterocytes are subject to constant turnover, undergoing continuous and rapid regeneration by intestinal stem cells (ISCs), which have the ability to self-renew and generate undifferentiated transit-amplifying (TA) cells in the process. TA cells then differentiate into various cell types, including absorptive enterocytes, goblet cells, enteroendocrine cells, Paneth cells, M cells and tuft cells (Noah et al., 2011), localization of which differ according to cell type. Although ISCs are located at the bottom of the crypt, most differentiated cells migrate up the crypt villus in the small intestine. In contrast, Paneth cells, which represent an essential component of the ISC niche, are also located at the bottom of the crypt (Meran et al., 2017).

The small intestine plays a major role in the absorption of orally administered drugs. This absorption is influenced by a number of factors, including the presence of drug-metabolizing enzymes and transporters, among which cytochrome P450 3A4 (CYP3A4) is the most abundant CYP enzyme not only in the liver but also in the small intestine. CYP3A4 is involved in the metabolism of up to 50% of all drugs, which can substantially affect the bioavailability of orally administered drugs (Zanger and Schwab, 2013). Additionally, efflux transporters, such as P-glycoprotein (P-gp) and breast cancer-resistant protein (BCRP), are also abundantly expressed in intestinal enterocytes and function as barriers against xenobiotics. These findings support critical involvement of the small intestine in the pharmacokinetics of orally administered drugs due to the presence of both drug-metabolizing enzymes and drug transporters. However, current methods lack appropriate cells necessary to evaluate intestinal pharmacokinetics. Although Caco-2 cells are the most commonly used model of intestinal enterocytes, the activities and expression levels of metabolic enzymes and transporters in Caco-2 cells differ from those of human intestinal enterocytes (Harwood et al., 2016; Nakamura et al., 2002). To more precisely predict the pharmacokinetics of orally administered drugs in the small intestine, the use of human primary small intestinal enterocytes can be valuable. Li et al. reported that cryopreserved human primary intestinal enterocytes have P450 and non-P450 drug-metabolizing enzyme activities and P450 inducibility, and suggested that they may be useful as an *in vitro* experimental model for the evaluation of intestinal pharmacokinetics (Li et al., 2018). However, it is difficult to obtain and culture human primary intestinal enterocytes in two dimensions for a long enough period to study their pharmacokinetics (Grossmann et al., 1998; Sträter et al., 1996). In addition, there are problems associated with the use of human primary intestinal enterocytes for drug screening. For instance, there is a limited supply of cells of the same batch because they cannot be proliferated with their functions. Furthermore, there is substantial variation between batches due to their different genetic and environmental backgrounds. Recent technological developments have allowed the growth of intestinal primary enterocytes in microfluidic organ-on-a-chip systems. For instance, Vernetti et al. showed the possibility of culturing primary enterocytes using the organs-on-a-chip system (Vernetti et al., 2017). However they are generally expensive, have low throughput and require handling skills.

In recent years, human induced pluripotent stem (iPS) cells have garnered increased attention due to their pluripotency associated with differentiation into any cell type, rendering them a potentially useful tool for drug discovery and development. We previously reported that enterocytes derived from human iPS cells are useful cells for pharmacokinetic studies (Kabeya et al., 2018; Kodama et al., 2016; Iwao et al., 2015, 2014); however, the process associated with their acquisition and culture is time and resource intensive. Moreover, obtaining a large supply is difficult.

As a solution to these issues, maintaining and culturing ISCs has been considered. However, it is difficult to simply cultivate ISCs alone, as they lose cellular stemness and proliferation potential with repeated passages and normally maintain stemness by utilizing a special niche environment localized near the crypt bottom. It was reported that use of three-dimensional (3D) cultures extended the period during which intestinal cells can be cultured (Jung et al., 2011; Sato et al., 2011, 2009). Moreover, the organoids in 3D cultures display a villus-like structure similar to intestinal tissue and contain several cells that are consistent with the crypt niche of the intestines (Sawant-Basak et al., 2018; Onozato et al., 2018; Tamminen et al., 2015; Foulke-Abel et al., 2014; Jung et al., 2011; Spence et al., 2011; Sato et al., 2011, 2009). Although stem cell characteristics can reportedly be maintained by mimicking the environment and structure of the living intestine, the exchange and passage of medium in 3D cultures are complicated. Additionally, because organoids are usually cultured in a Matrigel containing extracellular matrix, cellular passage and recovery are complicated, and their shape and size are varied. Furthermore, the use of Matrigel is unsuitable for large-scale cultures because of its gel form. The quantitative evaluation of intestinal absorption using 3D intestinal organoids is not very feasible because of the difficulty in accessing apical and basal compartments. Recently, Capeling et al. reported that organoids can be passaged and cultured using alternative methods to Matrigel, and some researchers have shown that organoids can be dissociated and seeded onto Transwell inserts (Capeling et al., 2019; Van der Hee et al., 2018; Múnera et al., 2017; Fernando et al., 2017). In addition, accessible organ-on-a-chip to both compartments has also been reported. However, the number of such reports is still low, and the function of these cells has not been sufficiently evaluated. These findings suggest that intestinal enterocytes with monolayers and two-dimensional (2D) culture are more suitable for quantitative pharmacokinetic and pharmacological evaluation.

In this study, in order to resolve these issues, we attempted to establish a new 2D culture method for maintaining human iPS-cell-derived ISCs capable of differentiation into enterocytes by using factors that enhance intestinal stemness and lineage. Additionally, we evaluated whether the resulting enterocytes demonstrated appropriate pharmacokinetic functions.

## RESULTS

### Schematic outline of the differentiation of human iPS cells into enterocytes

**Fig. 1.**
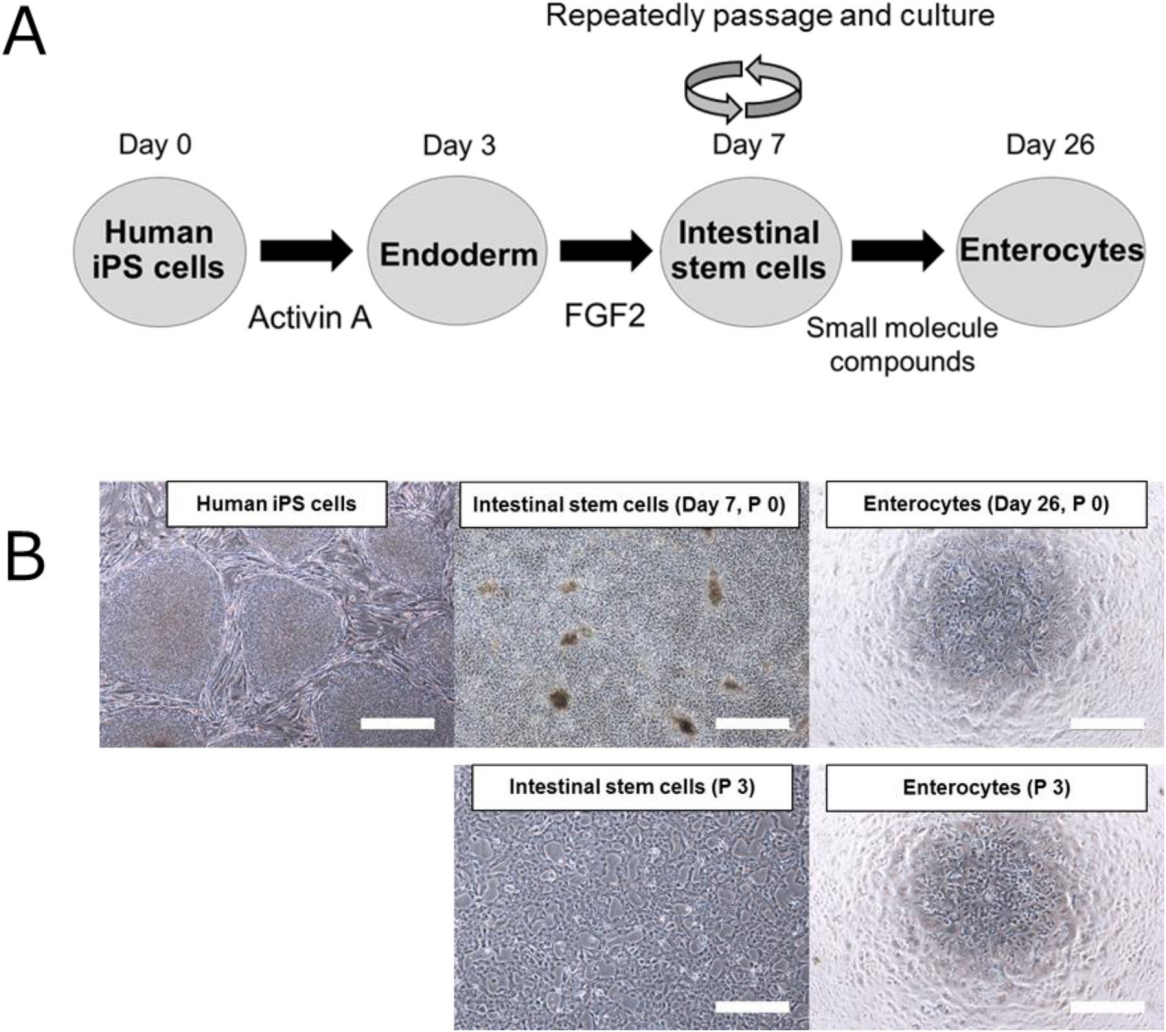
**Differentiation process of human iPS cells into enterocytes and associated morphological changes at each differentiation stage.** (A) Outline of the differentiation of human iPS cells into enterocytes. (B) Morphological images of human iPS cells, iPS-derived ISCs (P0 and P3) and enterocytes derived from human iPS cells (P0 and P3). Scale bars: 50 μm.

The process of human iPS cell differentiation into enterocytes is presented in Fig. 1A. Cells on day 7 after initiation of differentiation were established as ISCs. At this stage, we repeatedly passaged and cultured the cells on iMatrix-511-coated dishes using a medium containing several factors. ISCs from P0 to P7 were differentiated into enterocytes by changing to the differentiation medium. The morphologies of human iPS cells, ISCs (P0 and P3), and enterocytes (P0 and P3) are shown in Fig. 1B. No marked morphological difference was observed between P0 and P3 ISCs and enterocytes.

### Effects of foetal bovine serum (FBS) and KnockOut serum replacement (KSR) on mRNA levels of intestinal markers in ISCs derived from human iPS cells

**Fig. 2.**
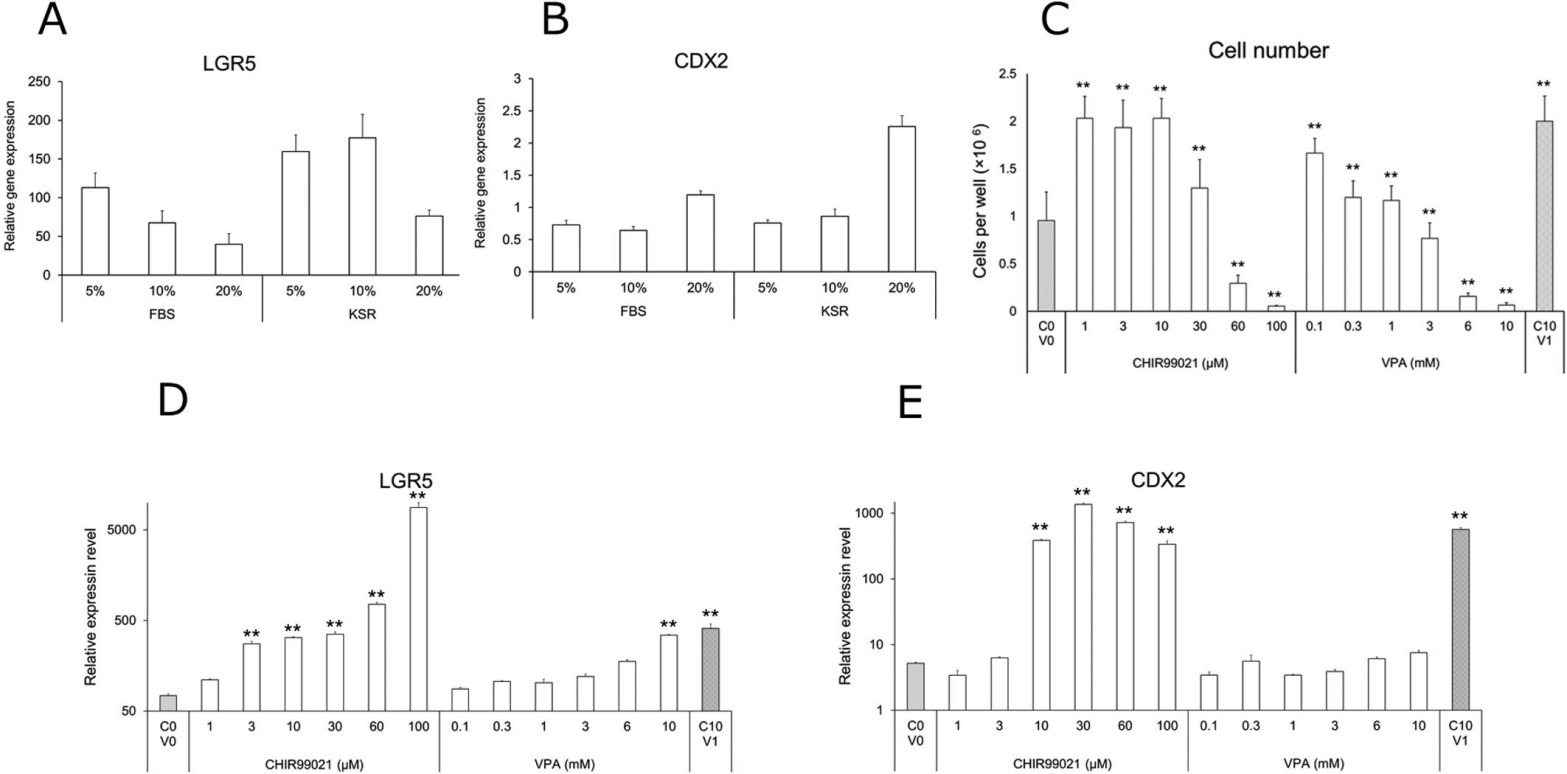
**Effect of FBS, KSR, CHIR99021 and VPA on CDX2 and LGR5 mRNA levels and cell growth in ISCs.** (A,B) Effect of FBS and KSR on CDX2 and LGR5 mRNA levels in ISCs derived from human iPS cells. LGR5 and CDX2 mRNA levels relative to levels in the human adult small intestine (set at 100). (C,D) Effect of CHIR99021 and VPA on CDX2 and LGR5 mRNA levels and cell growth in ISCs derived from human iPS cells. CDX2 and LGR5 mRNA levels are presented relative to levels in the human adult small intestine (set at 100). Cell number was counted using an automated cell counter. C0V0, concentrations of CHIR99021 and VPA (0); C10V1, concentrations of CHIR99021 and VPA (10 µM and 1 mM, respectively). Data represent the mean±s.d. (*n*=3); **P<0.01 compared with the C0V0.

Although FBS is widely used in cell-culture media, ISCs cannot be cultured in the presence of FBS, because it commonly contains growth factors and other proteins that can initiate their differentiation. KSR is a commercially available serum-free supplement often used to culture embryonic stem cells and in other serum-free cultures. Therefore, we evaluated the ability to substitute KSR for FBS in ISC cultures without affecting intestinal stemness and lineage (Fig. 2A,B).

We found that mRNA levels of the leucine-rich repeat-containing G protein-coupled receptor 5 (LGR5), an ISC marker, decreased along with increased FBS concentration. However, we observed a smaller decrease in LGR5 levels in the presence of KSR. Additionally, we observed no obvious differences in the mRNA levels of caudal type homeobox 2 (CDX2), an intestinal lineage marker, between FBS and KSR cultures. From these results, KSR appears to have less influence on cellular stemness and intestinal lineage when applied at a concentration of 10% to ISC cultures.

### Effects of CHIR99021 and valproic acid (VPA) on LGR5 and CDX2 mRNA levels in ISC

Many studies have reported that both WNT and Notch signalling are necessary for the maintenance of cellular stemness. Thus, we evaluated the influence of CHIR99021 and VPA on intestinal stemness and lineage in ISCs derived from human iPS cells. CHIR99021 inhibits GSK-3β-mediated β-catenin degradation and activates WNT signalling. VPA is a histone-deacetylase inhibitor that activates Notch signalling (Greenblatt et al., 2007), although the details of the underlying mechanism remain unclear. We found that CHIR99021 increased LGR5 and CDX2 mRNA levels in a concentration-dependent manner (Fig. 2C,D). VPA slightly increased LGR5 mRNA expression in a concentration-dependent manner, but had little effect on CDX2 mRNA expression at all concentrations. Quantification of changes in mRNA levels in the presence of combined 10 μM CHIR99021 and 1 mM VPA treatment revealed that LGR5 and CDX2 levels were 1.3- and 1.5-fold higher, respectively, than in the presence of CHIR99021 alone.

### Effects of CHIR99021 and VPA on ISC growth

We observed that the ISC number was approximately two-fold higher than that of the control in the presence of up to 10 μM CHIR99021 (Fig. 2E), whereas the cell number at 30 μM CHIR99021 was comparable to that of the control. Furthermore, the ISC number decreased with increasing concentrations of CHIR99021 and VPA and at concentrations >60 µM CHIR99021 or 3 mM VPA relative to the control. Combination treatment with 10 μM CHIR99021 and 1 mM VPA did not affect cell number relative to the results in the presence of 1 mM VPA alone. Although VPA had little effect on intestinal stemness and lineage, given that these results and previous reports support the importance of Notch signalling in cell stemness (Greenblatt et al., 2007), we conducted subsequent studies in the presence of 10 μM CHIR99021 and 1 mM VPA.

### Alterations in LGR5, Ki67, and CDX2 mRNA and protein levels according to ISC passage

**Fig. 3.**
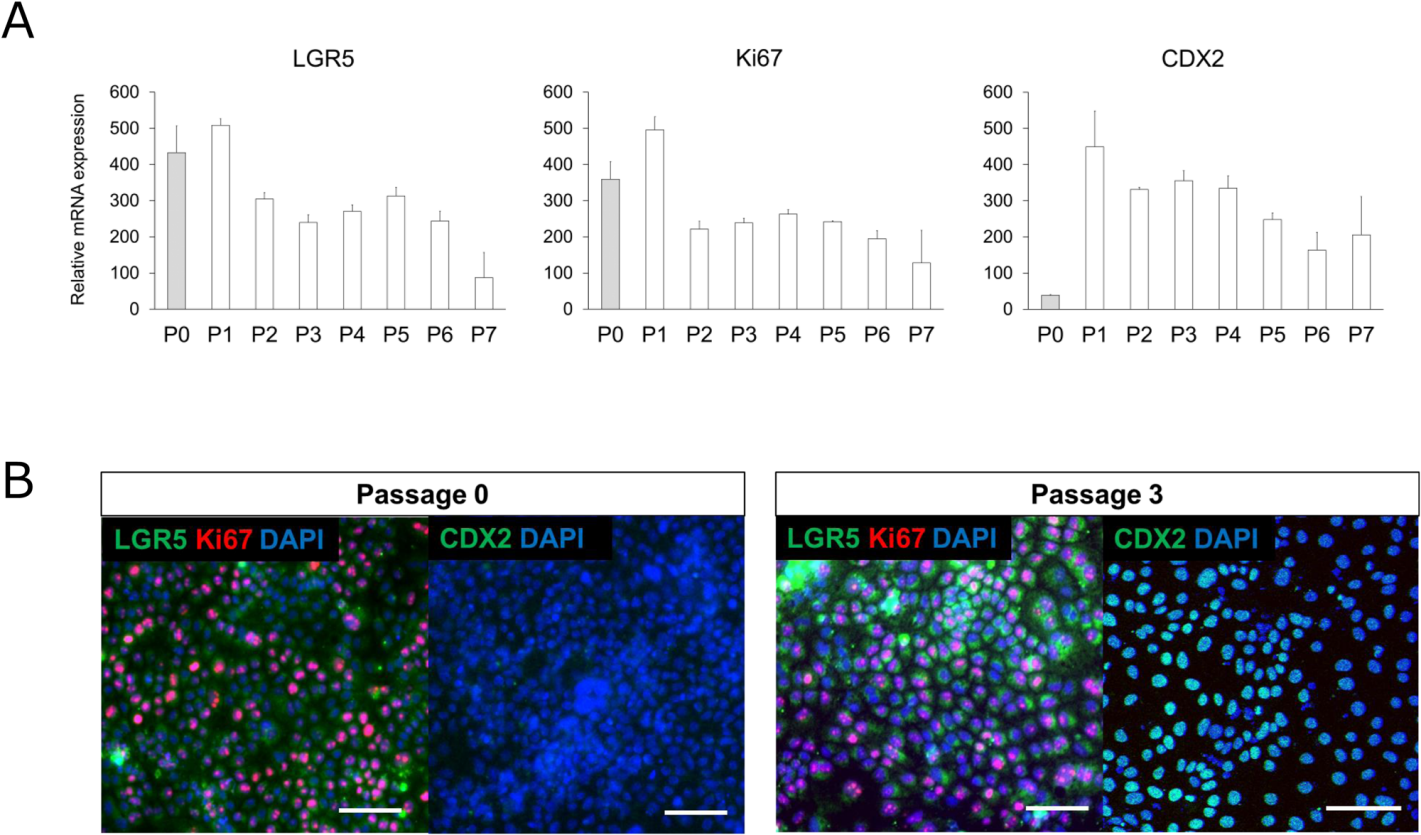
**Effects of passage number on LGR5, Ki67 and CDX2 mRNA levels and immunofluorescence analysis of LGR5, Ki67 and CDX2 in ISCs.** (A) Total RNA from repeatedly passaged ISCs (P0–P7) was extracted and gene expression levels were measured by real-time RT-PCR. The expression levels of each gene were normalized to those of HPRT1 and are presented relative to levels in the human adult small intestine (set at 100). Data represent the mean±s.d. (*n*=3). (B) ISCs (P0 and P3) were stained with LGR5 (green), Ki67 (red) and CDX2 (green), respectively. Scale bars: 100 μm.

To investigate whether repeated passage of ISCs maintained intestinal stemness and lineage, we evaluated mRNA and protein levels of LGR5, Ki67, and CDX2 (Fig. 3A,B). Ki67 is a stem cell and proliferation marker. Although the mRNA expression levels of LGR5 and Ki67 tended to decrease with passage, the expression levels were higher than or similar to those in human adult small intestine. In contrast, levels of CDX2 were higher in P1 to P7 relative to P0 and human adult small intestine. The fractional changes in LGR5, Ki67 and CDX2 expression levels in P1 to P7 relative to P0 are shown in Fig. S3. Immunofluorescence analysis detected LGR5 and Ki67 in P0 ISC whereas CDX2 was not detected, which is consistent with the low CDX2 mRNA levels observed at this passage. In contrast, LGR5, Ki67 and CDX2 were detected in P3 ISCs. Almost all cells were LGR5-, Ki67- and CDX2-positive in P3. These results show that intestinal stemness and lineage were maintained at the mRNA and protein levels.

### Alterations in mRNA and protein levels of enterocyte-differentiation markers according to ISC passage

**Fig. 4.**
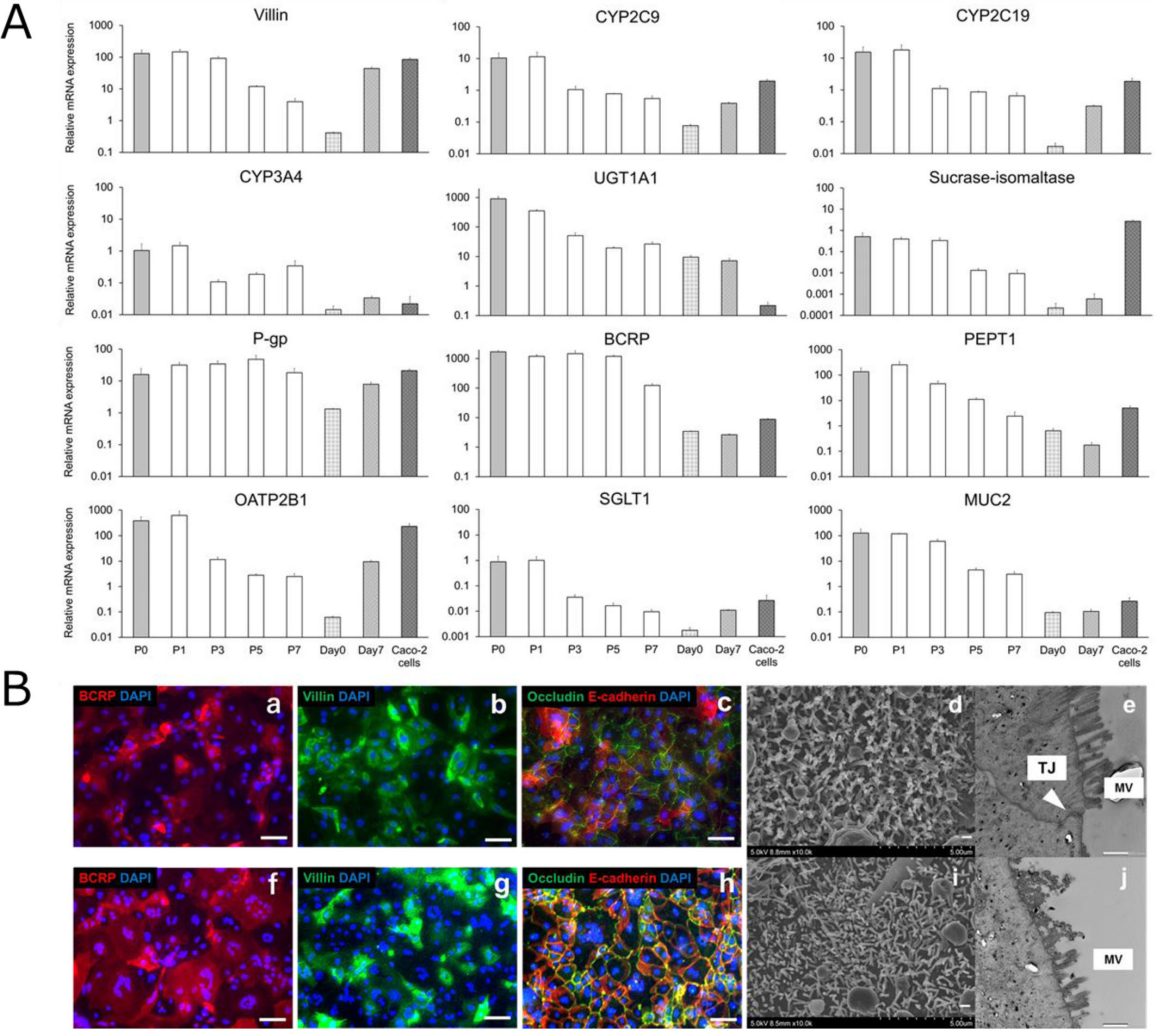
**Effects of passage number on ISCs differentiation into enterocytes.** (A) The expression levels of each gene were normalized to those of HPRT1 and are presented relative to levels in the human adult small intestine (set at 100). Data represent the mean±s.d. (*n*=3). (B) ISCs (P0 and P3) were stained for (a,f) BCRP, (b,g) villin and (c,h) occludin/E-cadherin. Scale bars: 100 μm. (d,e,i,j) SEM and TEM analyses of microvilli (MV) and (e) tight junctions. Scale bars: 500 nm. (d,i) Surface morphology of the enterocytes was visualized by SEM. Microvilli were detected in P0 and P3 enterocytes. (e,j) TEM analysis of enterocytes derived from human iPS cells and displaying enterocyte-like differentiation features. Tight junction was observed in P0 enterocytes.

To investigate whether the repeatedly-passaged ISCs maintained the potency to differentiate into functional enterocytes, we evaluated mRNA and protein levels of several enterocyte markers (Fig. 4A,B). The fractional changes in differentiation markers in P1–P7 enterocytes relative to P0 enterocytes (set at 100) are shown in Fig. S4. The mRNA level of BCRP was higher in P0–P7 enterocytes than in human adult intestine, and that of P-gp was almost maintained from P0–P7 enterocytes. Additionally, the levels of several markers – including villin, sucrase-isomaltase, and mucin 2 (MUC2) – in P3 enterocytes were similar to those in P0 enterocytes and showed a decreasing trend with repeated passage, as did other markers such as CYP2C9, CYP2C19, CYP3A4, UDP glucuronosyltransferase 1A1 (UGT1A1), peptide transporter 1 (PEPT1), organic anion transporting polypeptide 2B1 (OATP2B1), and sodium glucose transport protein 1 (SGLT1). To evaluate differentiation potency, we then performed an immunofluorescence analysis of protein levels of several differentiation markers, including BCRP, villin, occludin and E-cadherin. All four of these markers were detected in P0 and P3 enterocytes, with microvilli observed on the surface of each enterocyte by scanning electron microscopy (SEM). Tight junctions were also observed in P0 enterocytes.

### Induction of CYP3A4 and P-gp expression by rifampicin and 1α,25-dihydroxyvitamin D_3_ in enterocytes derived from human iPS cells

**Fig. 5.**
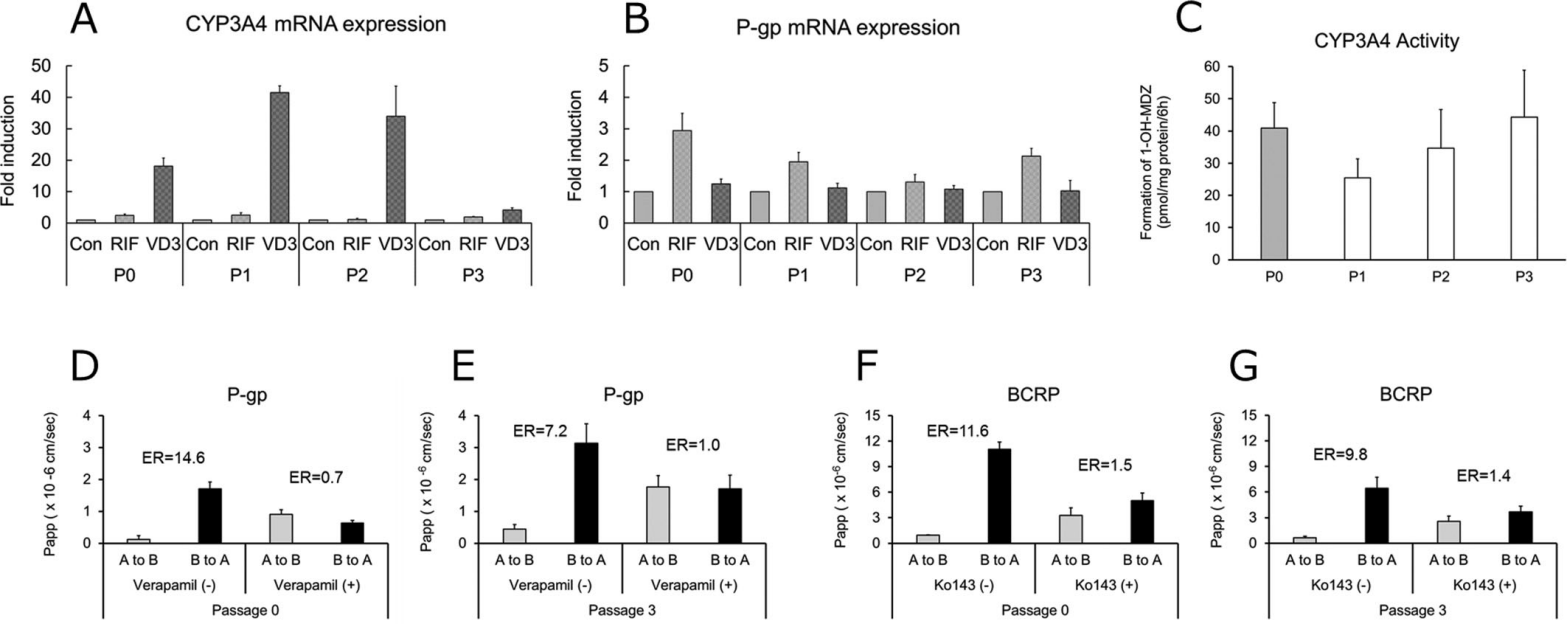
**Verification of inducibility and activity of metabolic enzyme and transporters in enterocytes.** (A) Induction of CYP3A4 and P-gp expression in enterocytes derived from human iPS cells. Target gene expression was normalized to HPRT1 levels. (B) Formation of 1′-hydroxy midazolam in enterocytes derived from human iPS cells. (C) Permeability of Rhodamine 123 and Hoechst 33342 across enterocyte monolayers. The efflux ratio (ER) for Rhodamine 123 or Hoechst 33342 was derived from the *P*_app_ values associated with basal-to-apical transport and apical-to-basal transport. Data represent the mean±s.d. (*n*=3).

We investigated whether CYP3A4 and P-gp expression was induced in P0 and P3 enterocytes (Fig. 5A,B). To assess the induction of CYP3A4 and P-gp expression, we used 30 μM rifampicin and 30 nM 1α,25-dihydroxyvitamin D_3_, ligands of the pregnane X receptor and vitamin D receptor, respectively. In P0 and P3 enterocytes, CYP3A4 mRNA levels ranged from 1.2- to 2.5-fold and 4.1- to 41.5-fold higher following rifampicin and 1α,25-dihydroxyvitamin D_3_ administration, respectively. Additionally, P-gp mRNA levels increased from 1.3- to 2.9-fold following rifampicin administration; however, levels showed only minimal increases following 1α,25-dihydroxyvitamin D_3_ administration.

### Formation of 1’-hydroxy midazolam in enterocytes

To evaluate CYP3A4 activity, enterocytes were incubated with 150 μM midazolam for 6 h and the formation of 1′-hydroxy midazolam, a specific metabolite of CYP3A4, was then measured. Metabolic activity was detectable in enterocytes derived from P0 to P3 ISCs, with levels of 1′-hydroxy midazolam formation comparable in P0 to P3 enterocytes (Fig. 5C).

### Permeability of Rhodamine 123 and Hoechst 33342 across enterocyte monolayers

P-gp and BCRP play an important role in limiting the intestinal absorption and tissue distribution of various drugs, and both transporters are highly expressed in the apical membrane of small intestinal enterocytes. Therefore, we conducted bidirectional-transport assays in order to examine enterocyte layers on the cell-culture inserts (Fig. 5D–G).

In the P-gp assays, the apparent permeability coefficient (*P*_app_) values of Rhodamine 123, a P-gp substrate, in the basal-to-apical direction were higher than those in the apical-to-basal direction in P0 and P3 enterocytes (Fig. 5D,E). The efflux ratio (ER), which is calculated from the directional ratio of *P*_app_, was 14.6 and 7.2 for P0 and P3, respectively. Following the addition of verapamil, a P-gp inhibitor, the basal-to-apical *P*_app_ values decreased, whereas the apical-to-basal *P*_app_ values increased along with a reduction in ER values to 0.7 and 1.0 in P0 and P3 cells, respectively.

In BCRP assays, the *P*_app_ values of the BCRP substrate Hoechst 33342 in the basal-to-apical direction were higher than those for the apical-to-basal direction in P0 and P3 enterocytes, with directional ERs of 11.6 and 9.8, respectively (Fig. 5F,G). Following addition of Ko143, a BCRP inhibitor, basal-to-apical *P*_app_ values decreased, whereas apical-to-basal *P*_app_ values increased along with reductions in ER to 1.5 and 1.4 in P0 and P3 cells, respectively. These results indicated that enterocytes derived from P0 and P3 membranes displayed P-gp and BCRP activity.

## DISCUSSION

The majority of drugs are absorbed in the small intestine, because the presence of villi and microvilli significantly increases the absorptive area. The intestine regulates the extent of orally administered drug absorption due to first-pass metabolism (Kharasch et al., 2003; Gibaldi et al., 1971). A number of metabolic enzymes, including CYP3A4 and transporters, such as P-gp and BCRP, are abundantly expressed in intestinal enterocytes, where they are heavily involved in drug metabolism and absorption. As a result, evaluation of intestinal pharmacokinetics is very important. Several studies have been undertaken with a focus on differentiating human iPS cells into intestinal cells (Negoro et al., 2018; Zhang et al., 2018; Ozawa et al., 2015; Ogaki et al., 2015), and the uses of intestinal cells have received increasing attention. We previously reported the usefulness of intestinal enterocytes derived from human iPS cells (Kabeya et al., 2018; Kodama et al., 2016; Iwao et al., 2015, 2014). Because enterocytes derived from human iPS cells express important metabolic enzymes and transporters, they serve as useful tools for evaluating pharmacokinetics in the intestinal tract. However, due to the time- and resource-intensive nature of experiments to differentiate human iPS cells into enterocytes, these issues must be addressed in order to increase the availability of these cells. In the present study, we established a new culture method for maintaining ISCs at the stage of differentiation from iPS cells and then evaluated whether the maintained ISCs retained the potential to differentiate into functional enterocytes.

We first examined the effect of FBS on ISC stemness. FBS is an essential supplement in cell culture and contains several hormone factors necessary for cell growth and proliferation. However, we previously reported that FBS promotes differentiation of human iPS cells into hepatocyte-like cells compared with results observed in the presence of KSR (Kondo et al., 2014). Notably, Ma et al. reported that FBS promotes murine corneal epithelial cell differentiation (Ma and Liu, 2011). In the present study, we observed decreased LGR5 mRNA levels with increased FBS concentration (Fig. 2A), whereas KSR did not affect these levels relative to FBS. Our findings suggest that KSR is suitable for culturing ISCs.

Maintenance of ISCs is difficult because the cells are located at the base of intestinal crypts and surrounded by a complex environment (i.e. the niche). Therefore, we used several factors (Table 1) involved in the niche to enhance or maintain ISC stemness. Among these factors, the most important was CHIR99021, an inhibitor of GSK-3β that reportedly improves stem cell properties by activating WNT signalling, which is critical for the maintenance of ISC stemness (Fevr et al., 2007). In the present study, CHIR99021 was shown to dramatically increase LGR5 and CDX2 levels (Fig. 2C,D). Notch signalling is also important for maintaining intestinal stem cell stemness (Demitrack and Samuelson, 2016; Horvay et al., 2013), and VPA represents an activator of Notch signalling (Greenblatt et al., 2008, 2007). Although VPA did not clearly affect LGR5 or CDX2 levels (Fig. 2C,D), an effect was observed when VPA was administered in combination with CHIR99021, resulting in maintenance of LGR5, Ki67 and CDX2 levels up to P7 (Fig. 3A). Moreover, we verified protein levels of LGR5, Ki67, and CDX2 in P3 ISC (Fig. 3B).

**Table 1.**
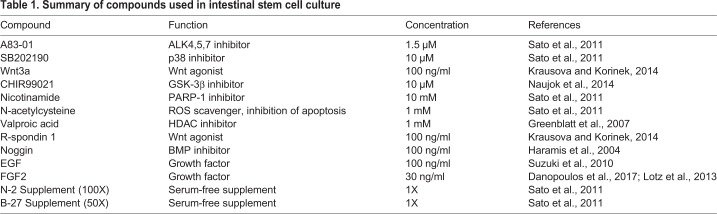
**Summary of compounds used in intestinal stem cell culture**

We then evaluated whether repeatedly passaged ISCs have the potential to differentiate into functional enterocytes. We verified that the mRNA levels of several differentiation markers were maintained after several passages (Fig. 4A), and that their protein levels were still observed in P3 enterocytes (Fig. 4B). We also confirmed the presence of microvilli, which are characteristic structures of the intestine and tight junctions in P0 enterocytes (although they were not observed in P3 enterocytes). These results indicate that repeatedly-passaged ISCs maintain the potential to differentiate into enterocytes.

Pharmacokinetic evaluation was performed using enterocytes derived from non-passaged and repeatedly-passaged ISCs. We observed similar CYP3A4 activity in P0 to P3 enterocytes and confirmed the induced expression of CYP3A4 by 1α,25-dihydroxyvitamin D_3_ and rifampicin and of P-gp by rifampicin in P0 and P3 enterocytes (Fig. 5A–C). Furthermore, P-gp and BCRP transport activities were detected in P0 and P3 enterocytes (Fig. 5D–G). Several studies have reported that metabolic enzymes and transporters, such as CYP3A4 and P-gp, in the small intestine work in coordination to reduce the intracellular concentration of drugs and, therefore, drug–drug interactions (DDIs) based on this interplay (Li et al., 2017; Zhang and Benet, 2001; Benet et al., 1999). Because few cells allow the simultaneous evaluation of metabolic enzymes and transporters, iPS-cell-derived enterocytes represent a promising model for evaluating the pharmacokinetics of the human small intestine and might better reflect enterocyte function than other model cells like Caco-2, HT-29, and LS-180 (Yamaura et al., 2016; Angelis and Turco, 2011; Walter et al., 1996). However, the activity of the CYP3A4 enzyme in enterocytes derived from human iPS cells was low compared to that in human primary enterocytes (Li et al., 2018). Although several studies have already reported methods for differentiating human iPS cells into enterocytes, these cells also reportedly express low levels of CYP3A4 enzyme compared with primary enterocytes. Therefore, further intestinal enterocyte maturation studies are required. Nonetheless, iPS-cell-derived enterocytes are considered useful as an *in vitro* model for the evaluation of intestinal pharmacokinetics and drug screening tool, because they are of non-carcinogenic origin and express more or equivalent intestinal markers, transporters and enzymes than Caco-2 cells. In addition, they show metabolic enzyme and transport activity and inducibility, and morphological feature such as microvilli. Induction of CYP3A by rifampicin has been reported in human intestinal enterocytes, while Caco-2 cells show no inducibility (Aiba et al., 2005; Glaeser et al., 2005; Kolars et al., 1992). Recently, several reports have suggested that intestinal cells can be maintained and cultured for a long period of time (Moorefield et al., 2018; Zhang et al., 2018; Wang et al., 2015). However, to the best of our knowledge, there are few reports evaluating intestinal pharmacokinetics in detail, including enzyme metabolic activity, inducibility and transport activity by using enterocytes derived from maintained ISCs. In the present study, CYP3A4-specific metabolic activity, transporter activity and inducibility of CYP3A4 and P-gp expression were observed in P0 and P3 enterocytes, with no notable differences between P0 and P3, except for a reduction in CYP3A4 inducibility. These results thus suggest that enterocytes, at least up to P3, are functional and suitable for pharmacokinetic evaluation. Further, they support the use of this method to reduce differentiation time and cost, as well as to supply sufficient quantities of enterocytes. For enterocytes after P3, further improvement of the maintenance of stem cells is necessary because the mRNA expression levels of differentiation markers were decreased relative to P0. In addition, the maintained ISCs after P3 showed cell detachment during the differentiation period, making it difficult to perform intestinal pharmacokinetics study. Furthermore, although we evaluated only CYP3A4 activity in the present study, other drug metabolizing enzymes such as carboxylesterase (CES), UDP-glucuronosyltransferase (UGT), sulfotransferase (SULT), N-acetyl transferases (NAT), flavin-containing monooxygenase (FMO) and monoamine oxidase (MAO) are also expressed in the small intestine and are involved in the intestinal metabolizing pathway. In our previous study, we reported that the patterns of CES expression and activity in the enterocytes were similar to those in the small intestine, and also reported activity of other CYP isoforms, UGT, and SULT (Kabeya et al., 2018, 2017). However, in addition to these enzymes, we also need to investigate expression and activity of enzymes such as NAT, FMO and MAO.

In summary, we established a method for culturing ISCs while maintaining cellular stemness. Our results showed that the differentiated enterocytes from the non-maintained and maintained ISCs demonstrated appropriate pharmacokinetic function. This culture method can potentially reduce the time and cost associated with generating large quantities of iPS-cell-derived enterocytes.

## MATERIALS AND METHODS

### Materials

Materials were purchased from the following sources: fibroblast growth factor (FGF) 2 and activin A (PeproTech, Rocky Hill, NJ, USA); iMatrix-511 (Nippi, Tokyo, Japan); CHIR99021 and Y-27632 (Focus Biomolecules, Plymouth Meeting, PA, USA); KSR, DMEM, DMEM/F-12, Advanced DMEM/F-12, N2 supplement and B27 serum-free supplement (Thermo Fisher Scientific, Waltham, MA, USA); FBS (Nichirei Biosciences, Tokyo, Japan); R-spondin 1, Noggin and epidermal growth factor (EGF) (GenScript, Piscataway, NJ, USA); N-acetylcysteine (Sigma-Aldrich, St Louis, MO, USA); 8-Br-cAMP (Enzo Life Sciences, New York, NY, USA); PD98059 and A-83-01 (AdooQ BIOSCIENCE, Irvine, CA, USA); 5-aza-2′-deoxycytidine (Chem-impex International, Wood Dale, IL, USA); L-glutamine, minimum essential medium nonessential amino acid solution (NEAA), penicillin-streptomycin solution, valproic acid and nicotinamide (Wako Pure Chemical Industries, Osaka, Japan); and total RNA from human small intestine samples (5 donors; BioChain Institute, Newark, CA, USA). All other reagents were of the highest quality available, including SB 202190 (ChemScene, Monmouth Junction, NJ, USA) and WNT3a (ATGen Co. Ltd., Gyeonggi-do, South Korea).

### Human iPS cell culture

Human iPS cells (Windy #51) were kindly provided by Dr A. Umezawa of the National Center for Child Health and Development (Tokyo, Japan). Human iPS cells were maintained on a feeder layer of mitomycin C-treated mouse embryonic fibroblasts with DMEM/F-12 supplemented with 20% KSR, 2 mM L-glutamine, 1% NEAA, 0.1 mM 2-mercaptoethanol ,and 5 ng/ml FGF2. The medium was changed daily. Since mouse embryonic fibroblasts cannot survive for a long period due to mitomycin C treatment, they were considered to be dead in the process of repeated passage of ISCs or differentiation period into enterocytes.

### Differentiation of human iPS cells into ISCs

Human iPS cells were differentiated into endoderm by the addition of 100 ng/ml activin A for 3 days and then differentiated into ISCs by 250 ng/ml FGF2 for 4 days. We established the cells as ISCs on day 7 after the initiation of differentiation. The schematic outline of the protocol for the differentiation of human iPS cells into ISCs is shown in Fig. S1.

### Evaluation of the effect of FBS and KSR on intestinal stemness and lineage and ISC lineage

After differentiation of the human iPS cells into ISCs, we passaged the ISCs on iMatrix-511-coated 24-well plates and cultured the cells with different concentrations of FBS or KSR for 3 days. After completion of the culture, the cells were collected, and LGR5 and CDX2 mRNA levels were measured.

### Evaluation of the effect of CHIR99021 and VPA on intestinal stemness and ISC growth and lineage

After the differentiation of human iPS cells into ISCs, we passaged the ISCs on iMatrix-511-coated 24-well plates and cultured the cells with different concentrations of CHIR99021 or VPA for 3 days. After completion of the culture, cells were collected, LGR5 and CDX2 mRNA levels were measured, and the number of cells was counted. Controls were treated with dimethyl sulfoxide.

### Culture of ISCs derived from human iPS cells

Human iPS cells were induced into ISCs, and the cells were passaged on iMatrix-511-coated dishes in culture medium (Advanced DMEM/F-12 supplemented with several factors shown in Table 1). The effect of these supplements is shown in Table 1. For cell passage, Y-27632 (10 μM) was added in the first 24 h, and the medium was subsequently changed every 2–3 days.

### Differentiation of ISCs into enterocytes

ISCs (P0–P7) were differentiated into enterocytes based on the methods described in our previous report. In brief, the maintained ISCs were passaged on iMatrix-511-coated 24-well plates or cell-culture inserts and cultured with medium containing EGF and 8-Br-cAMP. After 7 days of differentiation, PD98059, 5-aza-2′-deoxycytidine, A83-01, and 3-isobutyl-1-methylxanthine were added to differentiate into enterocytes. Additionally, we added 10 ng/ml FGF2 throughout the differentiation period. The medium was subsequently changed every 3 days. The schematic outline of the protocol for the differentiation of ISCs into enterocytes is shown in Fig. S2.

### RNA extraction, reverse transcription polymerase chain reaction (RT-PCR) and real-time PCR analysis

Total RNA was extracted with the Agencourt RNAdvance tissue kit (Beckman Coulter, Brea, CA, USA). First-strand cDNA was prepared from total RNA, and the reverse transcription reaction was conducted using ReverTra Ace qPCR RT master mix (TOYOBO, Shiga, Japan). Real-time PCR was conducted using an Applied Biosystems 7300 real-time PCR system with SDS software (v1.4; Applied Biosystems, Foster City, CA, USA). PCR was performed with the primer pairs listed in Table 2 using a KAPA SYBR Fast qPCR kit (NIPPON Genetics, Tokyo, Japan). The mRNA expression level was normalized relative to that of the housekeeping gene hypoxanthine phosphoribosyltransferase 1 (HPRT1). Gene expression levels in adult small intestine were defined as 100.

**Table 2.**
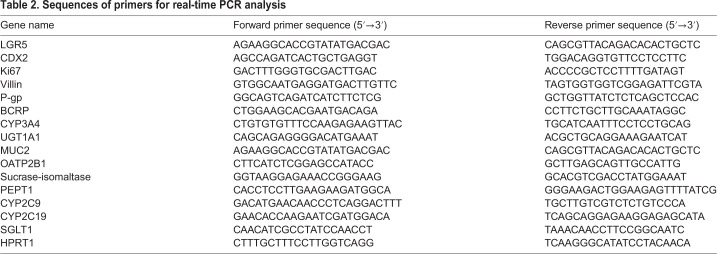
**Sequences of primers for real-time PCR analysis**

### Immunofluorescence staining

IPSCs (P0 and P3) and enterocytes differentiated from ISCs (P0 and P3) were washed with phosphate-buffered saline (PBS) and fixed and permeabilized with cold methanol for 5 min at 4°C. After washing with PBS, the cells were blocked in PBS containing 0.5% FBS for 20 min at room temperature. After the blocking step, the cells were incubated with primary antibody at room temperature for 60 min and subsequently incubated overnight at 4°C. The cells were then washed with PBS and incubated with secondary antibody for 60 min at room temperature. The antibodies used and their dilutions are shown in Table 3. After washing with PBS, the cells were incubated with 1 μg/ml of 4′,6-diamidino-2-phenylindole for 5 min at room temperature, followed by washing with PBS. The cells were analysed using an ECLIPSE Ti-S microscope (Nikon, Tokyo, Japan).

**Table 3.**
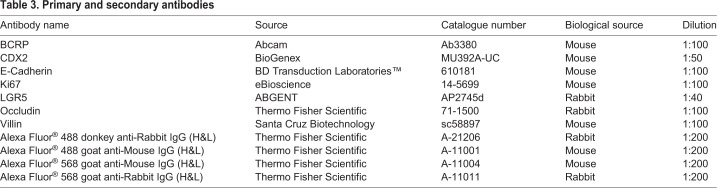
**Primary and secondary antibodies**

### Transmission electron microscopy (TEM)

Enterocytes differentiated from ISCs (P0 and P3) were fixed with 2.5% glutaraldehyde overnight at 4°C and post-fixed with 1% osmium tetroxide for 2 h at 4°C. Subsequently, the samples were dehydrated with ethanol and embedded in resin. Then, embedded samples were cut into 0.1-µm sections, which were stained with uranyl acetate and observed using a Hitachi H7600 transmission electron microscope (JEOL, Tokyo, Japan).

### Scanning electron microscopy (SEM)

Enterocytes differentiated from maintained ISCs (P0 and P3) were fixed and dehydrated as described for TEM preparations. Subsequently, the samples were dehydrated with ethanol and critical point drying was performed using a CPD 300 (Leica, Wetzlar, Germany). Osmium coating was performed with a plasma coater (OPC80AJ; Filgen, Nagoya, Japan). Images of the fractured surface of the freeze-dried samples were obtained using an S-4800 microscope (Hitachi, Tokyo, Japan).

### CYP3A4 and P-gp induction assays

Enterocytes differentiated from ISCs (P0 and P3) were treated with 30 nM 1α,25-dihydroxyvitamin D_3_ or 30 μM rifampicin, known inducers of CYP3A4 and P-gp expression, respectively, for 48 h. Controls were treated with dimethyl sulfoxide. Inducibility was evaluated by quantifying the mRNA levels of CYP3A4 and P-gp.

### Determination of CYP3A4 activity

Enterocytes differentiated from ISCs (P0 and P3) were incubated with Hanks' balanced salt solution (HBSS) containing 150 µM midazolam for 6 h at 37°C. After incubation, 200 µl of the reaction medium was collected and added to 200 µl of ice-cold acetonitrile containing 3.6 µM chlorpropamide as an internal standard. Enterocytes were lysed with 150 µl of 1 M NaOH, with an equal amount of 1 M HCl added for neutralization. The protein content was then measured with a Pierce BCA protein assay kit (Thermo Fisher Scientific). The formation of 1′-hydroxylated metabolites was measured using ultra-performance liquid chromatography–tandem mass spectrometry (UPLC–MS/MS) system (ACQUITY UPLC^®^ System and Micromass^®^ Quattro Premier™ XE, Waters, Milford, MA, USA) equipped with an XBridge BEH C18 column (2.1×50 mm, 3.5 µm; Waters). Samples were centrifuged at 20,600* **g*** for 5 min at 4°C. Ten microliters of supernatant were injected into the UPLC-MS/MS system. The mobile phase comprised 10 mM ammonium formate (A) and acetonitrile (B), and the sample and column temperatures were 4°C and 40°C, respectively. The following gradient program was used: 0–0.5 min: 5% B, 0.5–2.0 min: 5→95% B (linear), 2.1–3.5 min: 95% B, 3.6–5.4 min: 95→5% B (linear). The flow rate was set at 0.25 ml/min (except for 2.1–3.5 min; 0.55 ml/min). Multiple-reaction monitoring in positive ion electrospray was used for the detection of 1′-hydroxy midazolam derivatives (*m/z* 342.03→168.40). All data were analysed with Mass Lynx 4.1 software (Waters).

### Transport study of Rhodamine 123

ISCs (P0 and P3) were seeded on the inserts of Corning Transwells (Corning, NY, USA) and cultured for 19 days. After pre-incubation with HBSS (pH 7.4) for 20 min, Rhodamine 123 solution was added to the apical side to a final concentration of 1 μM and then drawn out of the basal side at 30-min intervals for up to 120 min at 37°C in the presence or absence of 30 µM of the P-gp inhibitor verapamil. An equal volume of HBSS was immediately added to the basal side after each sampling. Fluorescence was measured using a SYNERGY HTX system at 505 nm excitation and 534 nm emission (Bio-Tek, Winooski, VT, USA). *P*_app_ was calculated using the following equation:
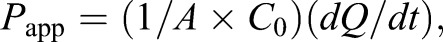
where d*Q*/d*t* is the rate of drug permeation across the cell monolayer, *C*_0_ is the donor-compartment concentration at time zero and *A* is the area of the cell monolayer. The ER was defined as *P*_app_ (basal-to-apical)/*P*_app_ (apical-to-basal).

### Transport study of Hoechst 33342

We used the same methods as those described for Rhodamine 123 transport to analyse the transport of Hoechst 33342. Hoechst 33342 was used at a final concentration of 20 μM. Ko143 was used as BCRP inhibitor at a final concentration of 1 µM. Fluorescence was measured at 352 nm excitation and 461 nm emission, and *P*_app_ was calculated.

### Statistical analysis

Statistical significance was assessed with Student's *t*-tests for analysis of variance, followed by Duncan's new multiple range test. Data are represented as the mean±s.d. from three biological replicates. Significant differences are indicated by *P*-values listed in the figure legends. A *P*-value <0.05 was considered to be statistically significant.

## Supplementary Material

Supplementary information
