# Supplementary material for: Establishment of a novel culture method for maintaining intestinal stem cells derived from human induced pluripotent stem cells
Source: Biol Open. 2020 Jan 9;9(1):bio049064. doi: 10.1242/bio.049064 (PMC6955217; doi:10.1242/bio.049064)
Supplement: Supplementary information [file biolopen-9-049064-s1.pdf]

Figure S1. Schematic of the protocol for the differentiation of human iPS cells into intestinal stem cells.

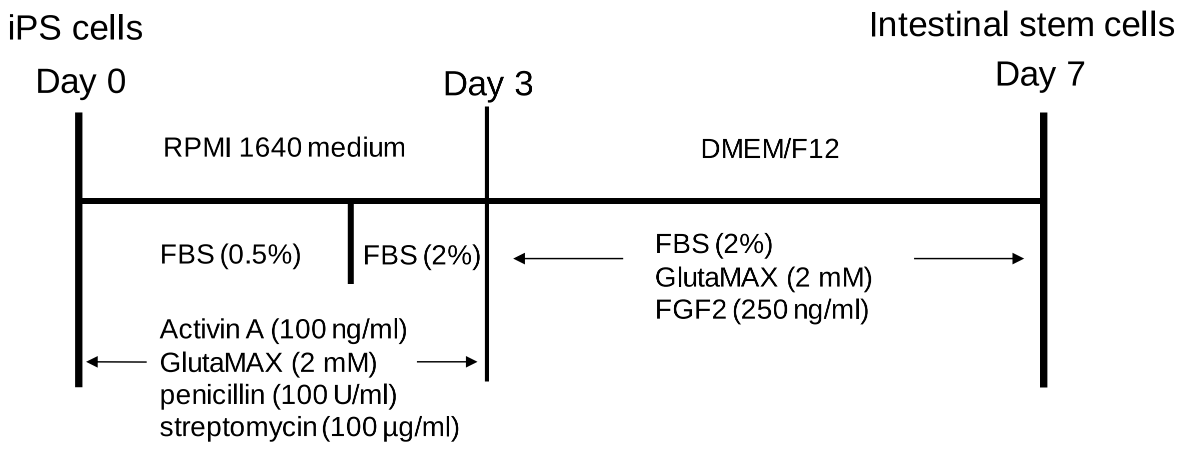

Biology Open • Supplementary information

Figure S2. Schematic of the protocol for the differentiation of intestinal stem cells into enterocytes.

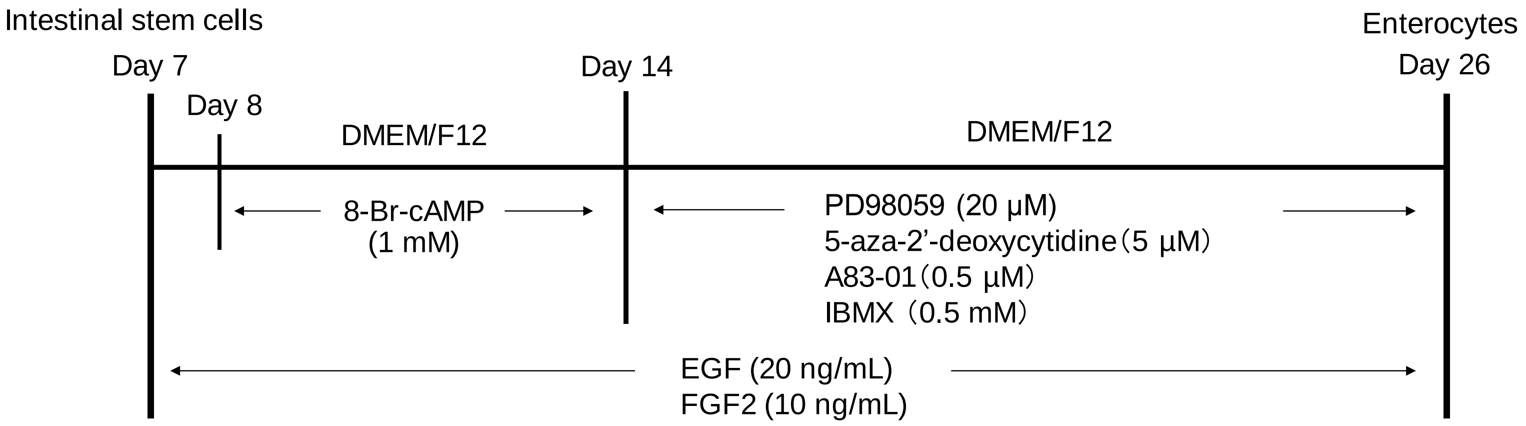

Biology Open • Supplementary information

Figure S3. Fractional changes of mRNA expression levels of differentiation markers.

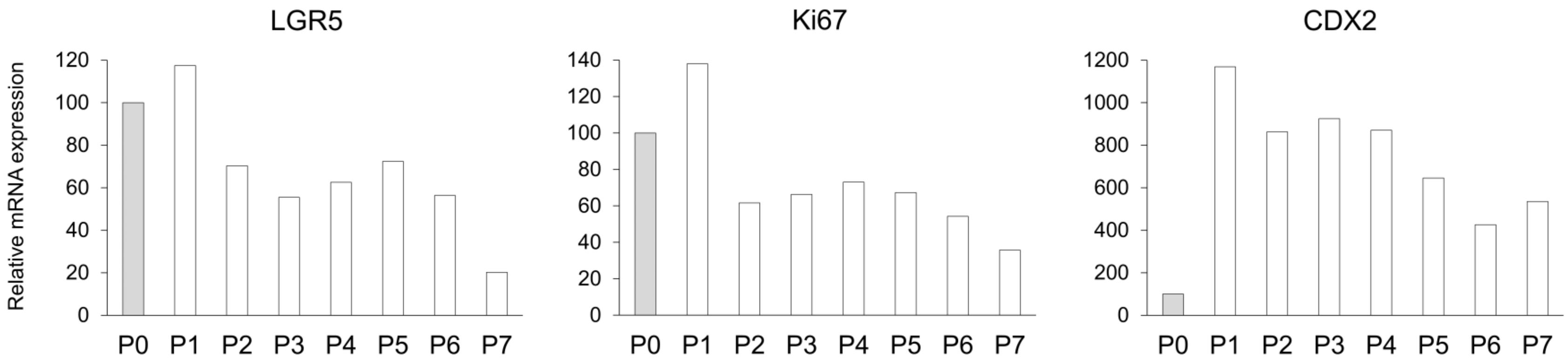

Biology Open • Supplementary information

Figure S4. Fractional changes of mRNA expression levels of intestinal markers and pharmacokinetic-related genes.

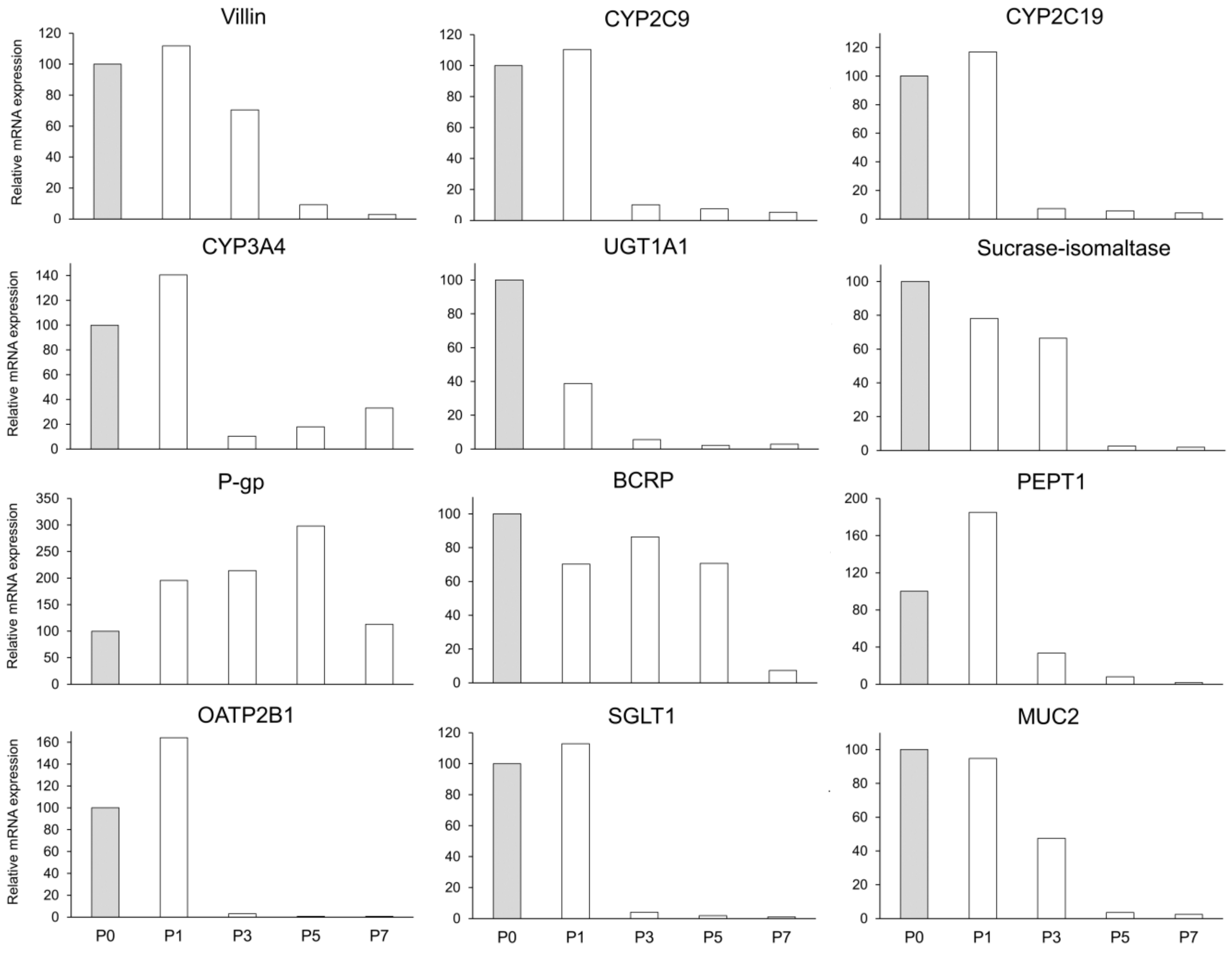

Biology Open • Supplementary information
